# Network meta-analysis of different liver protective drugs in the treatment of drug-induced liver injury

**DOI:** 10.1097/MD.0000000000036538

**Published:** 2023-12-15

**Authors:** Chengcheng Li, Xin Yang, Yuhang Quan, Anhao Wu, Yifan Wang

**Affiliations:** a Department of Thyroid and Breast Surgery, The First People’s Hospital of Honghe State, Mengzi, China; b Department of Blood Transfusion, The First People’s Hospital of Yunnan Province, The Affiliated Hospital of Kunming University of Science and Technology, Kunming, China; c Department of Anesthesiology, The Third Affiliated Hospital of Kunming Medical University, Yunnan Cancer Hospital, Yunnan Cancer Center, Kunming, China; d Department of Mammary Surgery I, The Third Affiliated Hospital of Kunming Medical University, Yunnan Cancer Hospital, Yunnan Cancer Center, Kunming, China.

**Keywords:** drug-induced liver injury, liver protective drugs, network meta-analysis, treatment measures

## Abstract

**Background:**

Currently, drug-induced liver injury (DILI) has become one of those public issues in society, which has added a huge burden to both the individuals and the society. In the current clinical stage, there are numerous drugs developed to treat this disease, and different drug treatment measures have been proven to achieve certain clinical efficacy in the corresponding randomized controlled trials. However, there are still many therapeutic drugs that have not been directly compared and studied. Therefore, it is difficult to directly compare the effectiveness and safety of various strategies for the treatment of DILI. In this regard, the present study collected the therapeutic efficacy of diverse treatments in DILI in recent years through network meta-analysis, evaluated and screened the existing optimal clinical therapeutic plan, and helped physicians formulate clinical therapeutic plans.

**Methods:**

Databases, including the Chinese Journal Full-text Database, Wanfang Data Journal Paper Resources (Wangfang), VIP Chinese Science and Technology Journal Full-text Database, The Cochrane Library, PubMed, and EMBASE, were searched using keywords from inception to January 2023. Eligible randomized controlled trials were selected in line with eligibility criteria, and mesh meta-analysis of binary variables was carried out using Stata 16 software.

**Conclusion:**

In combination with alanine aminotransferase, aspartate aminotransferase, and total bilirubin, MI may be the intervention measure for minimizing alanine aminotransferase levels in patients after treatment. Besides, compound glycyrrhizin may be the intervention for minimizing aspartate aminotransferase levels in patients after treatment, and polyene phosphatidylcholine may be the intervention for minimizing total bilirubin levels in patients after treatment. Placebo is the potential intervention that has the least adverse reactions post-treatment, and RT has the second least adverse reactions. Moreover, hepatocyte growth-promoting factors may be the most effective intervention after treatment.

**Results:**

To sum up, the present work compared the clinical effects of 13 liver protective drugs through meta-analysis and provided a systematic understanding of commonly used drugs for the treatment of DILI in clinical practice.

## 1. Introduction

Drug-induced liver injury (DILI), the liver dysfunction resulting from drugs or herbal medicines, is usually diagnosed following the exclusion of additional causes.^[1,2]^ In China, the incidence rate of DILI is 23.8/100000, higher than that in Western countries, and it shows a rapidly increasing trend.^[3,4]^ According to the pathogenesis, DILI is generally divided into 3 categories, namely, direct hepatotoxicity, idiosyncratic hepatotoxicity (heterogeneous), and indirect liver injury. Direct hepatotoxicity refers to the direct damage to the liver caused by the drug itself or its metabolites and is related to the drug dosage. Heterogeneous hepatotoxicity is not related to the drug itself; instead, it is mostly associated with the heterogeneity of the body. Due to the hypersensitivity or reduced tolerance to drugs of the special constitution, the accumulation of drugs or intermediate products can lead to liver damage. Indirect liver injury is commonly seen in tumor patients who receive targeted immunotherapy, and the drugs can induce or worsen the underlying liver diseases by altering the immune function of the body.^[5]^ Most acute DILI patients do not show any obvious symptoms, only the increased levels of liver biochemical indicators such as alanine aminotransferase (ALT), aspartate aminotransferase (AST), alkaline phosphatase, and gltamyltranspeptidase in serum to varying degrees. At present, the drugs used to treat DILI in clinical practice are roughly divided into 6 categories, including anti-inflammatory drugs, liver cell membrane repair protectors, detoxification drugs, antioxidant drugs, cholagogic drugs, and drugs that promote liver regeneration. Each type contains one to several representative drugs, which can be used alone or in combination.^[6]^ Research has shown that each drug has a certain therapeutic effect and corresponding side effects, but currently, there are still relatively few drugs in randomized controlled trials (RCTs) that focus on treating DILI, and research progress in treating acute DILI is slow. The present study collected the therapeutic efficacy of diverse treatments in DILI in recent years through network meta-analysis, evaluated and screened the existing optimal clinical therapeutic plan, and helped physicians formulate clinical therapeutic plans.

## 2. Data and methods

### 2.1. Data

Databases including VIP Chinese Science and Technology Journal Full-text Database, Chinese Journal Full-text Database, The Cochrane Library, Wanfang Data Journal Paper Resources (Wangfang), EMBASE, and PubMed were systemically searched using keywords from inception to January 2023. Retrieval formulas utilized were shown below, theme: hepatoprotective or liver protection or Reduced glutathione or tiopronin or bicyclol or Silymarin or Ursodeoxycholic Acid or Cholestyramine or Polyene phosphatidylcholine or Glycyrrhizic acid or glycyrrhizin(snmc) or Magnesium Isoglycyrrhizinate or diammonium glycyrrhizinate or ammonium glycyrrhizinate or hepatocyte growth promoting factor, and the theme: Drug-induced liver injury.

Eligible RCTs were selected in line with eligibility criteria.

Studies below were included: RCTs; studies published in Chinese or English language; no restrictions on age, gender, race, or disease course in patients, but baseline comparability was required; the interventions in the study were different liver protective drugs; the liver injury in the patient was consistent with the diagnosis of DILI^[7]^; and the study outcome indicators included ALT, AST, adverse reactions, effective rate, together with total bilirubin (TBIL). Treatment is effective refers to the improvement of clinical symptoms and signs in patients and a 50% decrease in liver function indicators compared to before treatment.

Studies below were excluded: duplicates and the original text were not found; overviews, experience summaries, case reports, meeting abstracts, and meta-analyses; disease diagnosis did not match with DILI; and the intervention measures included the combined use of multiple (≥2) liver protective drugs.

### 2.2. Protocol registration

The present work was conducted in strict accordance with the preferred reporting items of the system review and meta-analysis program (PRISMA-P),^[8]^ and registered at the INPLASY website (registration number: INPLASY202360039). Any adjustments in the study will be updated timely in our eventual article.

### 2.3. Methods

Those obtained studies were imported into the endnote software, and duplicates were removed, for the rest of the studies, nonqualified ones were removed after title and abstract reading in line with our predetermined eligibility criteria. Thereafter, 2 reviewers read full texts of the rest studies, and those with duplicated data, insufficient data, or with no outcome indicators were further eliminated. To ensure data accuracy and research precision, related data were obtained, including author, publication year, baseline data, intervention measures, together with outcome indicators. The collected data were combined and further examined. Any dispute between them was solved by the opinion of an experienced third reviewer.

The RCT evaluation tool from the Risk of Bias 2 (RoB 2) tool developed by Cochrane.

### 2.4. Statistical analysis

Mesh meta-analysis of binary variables was conducted with Stata 16. Overall consistency of indirect with direct evidence was analyzed through the inconsistency test. *P* > .05 stood for the absence of consistency, as a result, fitting of the consistency model should be conducted, otherwise, fitting of the inconsistent model should be carried out. Additionally, this work utilized the node-splitting approach for testing local inconsistency in indirect compared with direct comparisons, where *P* < .05 indicated local inconsistency. Enumeration data were represented by relative risks as well as relevant 95% confidence intervals (CIs). Moreover, intervention measure efficacy was sorted out in line with the area under the cumulative probability (surface under the cumulative ranking, SUCRA), with the greater area under the curve indicating superior intervention measure efficacy. If there were over 10 studies incorporated into the outcome index, the distribution symmetry of points was observed on the funnel chart to assess publication bias.

### 2.5. Ethics and dissemination

Data in the present systematic review and network meta-analysis were obtained in published studies, and no human being was involved, therefore, it was unnecessary to receive ethical approval. Our results shed more light on peer reviews and may be displayed during related meetings.

## 3. Results

### 3.1. Study selection

**Figure 1.**
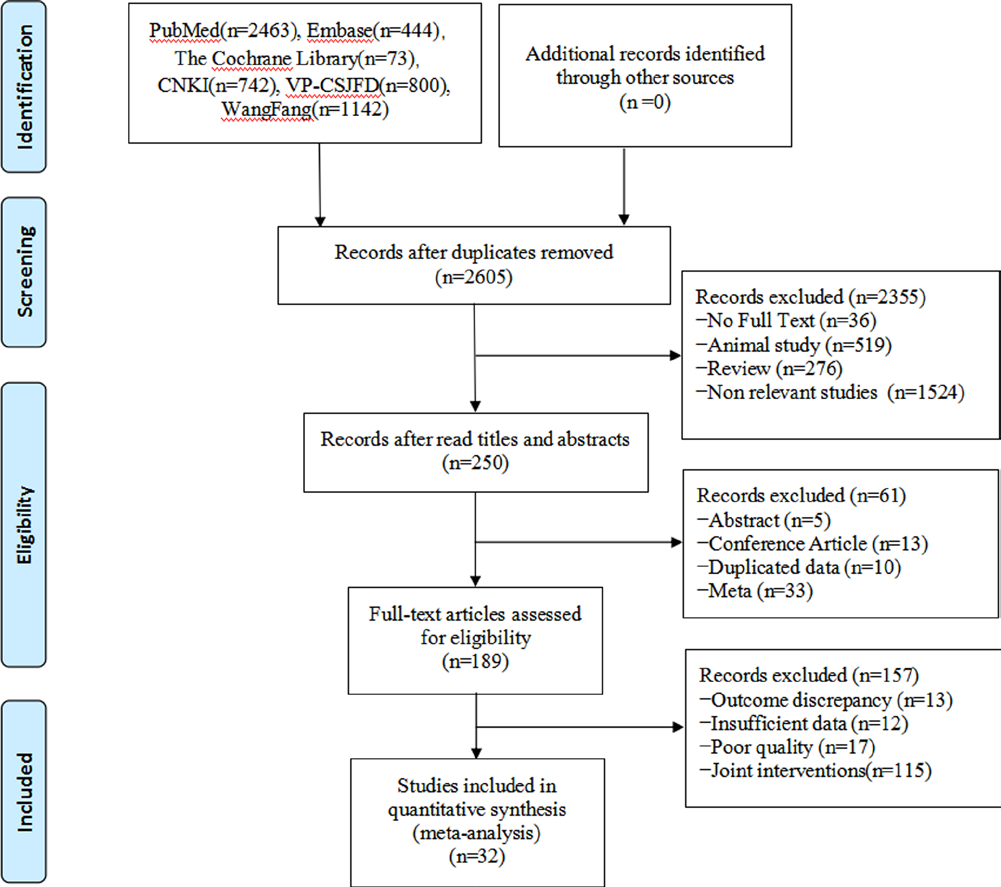
Flow chart of literature screening.

Altogether, 5664 studies were selected by the systemic search, among which, 2650 were retained following the removal of duplicates with Endnote X9 software. Following title and abstract reading, we eliminated irrelevant articles, meta-analyses, abstracts, conferences, and noncontrolled experimental studies to obtain 250 qualified studies. Full texts of the rest of the studies were carefully read. At last, 32 studies involving 3026 cases were enrolled^[9–41]^ (Fig. 1).

### 3.2. Study quality

**Figure 2.**
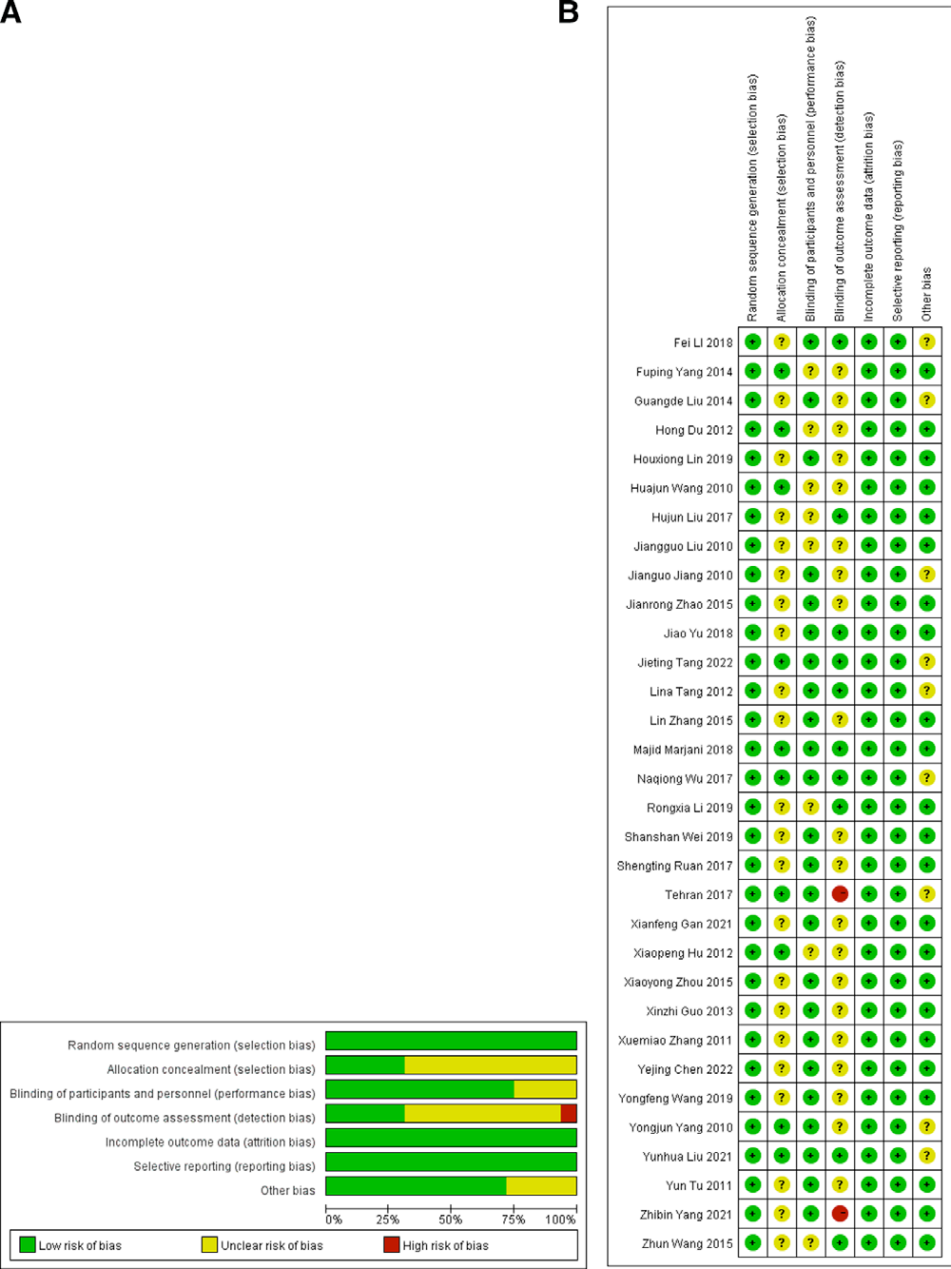
(A) Risk of bias graph. (B) Risk of bias summary.

The basic information of the included studies is presented in Table 1. Figure 2A shows that the included literature only has high-risk evaluation items on measurement bias, mainly due to unclear allocation of hidden schemes, whether double blind, triple blind, and other biases are used, and the overall quality of the literature is acceptable. The results in Figure 2B show that only 2 articles have high-risk evaluation items for measurement bias, but the results of these 2 articles on other evaluation items show that the quality of these 2 articles is good. All the articles mainly have an unclear allocation of hidden schemes, whether to use a double blind or triple blind, and the overall quality of the literature is acceptable.

**Table 1:** The basic characteristics and methodological quality assessment of included studies.

Author	Country	Year	Research type	Interventions		Sample size		Age (year)		Sex (M/F)			Diagnostic criteria
				Observation group	Control group	Observation group	Control group	Observation group	Control group	Observation group		Control group	
Hujun liu	China	2017	RCT	PPPC	RG	22	22	51.3 ± 4.2	51.5 ± 4.3	13/9	12/10		Undescribed
					Tiopronin		22		51.8 ± 4.3		14/8		
Zhun Wang	China	2015	RCT	RG	PPPC	30	30	32.65 ± 4.29	32.68 ± 4.27	17/13	18/12		Undescribed
					Tiopronin		30		32.69 ± 4.25		19/11		
Hong Du	China	2012	RCT	RG	CG	42	42	44.6 ± 8.7	47.3 ± 10.2	26/16	22/20		Undescribed
Rongxia Li	China	2019	RCT	RG	CGAM	53	53	49 ± 6	49 ± 5	17/36	16/37		The judgment method 1
					HGPF		53		49 ± 6		17/36		
Jiao Yu	China	2018	RCT	PPPC	Tiopronin	45	45		48.2 ± 6.9	51/39			The judgment method 2
Yunhua Liu	China	2021	RCT	PPPC	DG	39	39	50.7 ± 5.0		42/36			The judgment method 2
Fuping Yang	China	2014	RCT	PPPC	Tiopronin	42	41	50.32 ± 7.14	49.26 ± 6.48	22/20	21/20		Undescribed
Huajun Wang	China	2010	RCT	CG	RG	29	29	26–69		44/21			Undescribed
Xianfeng Gan	China	2021	RCT	PPPC	DG	60	60	51 ± 5	50 ± 5	34/26	33/27		The judgment method 2
Yongjun Yang	China	2010	RCT	Tiopronin	RT	46	46	43		78/14			Undescribed
Jianguo Liu	China	2010	RCT	BA	DG	60	60	46.45 ± 11.68	49.94 ± 13.31	20/10	37/23		The judgment method 1
Fei Li	China	2018	RCT	BA	DG	49	49	46.58 ± 5.48	46.27 ± 5.55	25/24	29/20		Undescribed
Houxiong Lin	China	2019	RCT	BA	PPPC	62	74	40.7 ± 7.2		78/58			The judgment method 1
Xiaoyong Zhou	China	2015	RCT	BA	DG	52	51	43	41	31/21	32/19		The judgment method 3
Yun Tu	China	2011	RCT	BA	DG	77	76	51.4		101/52			The judgment method 4
Lin Zhang	China	2015	RCT	BA	Silymarin	61	51	31.9 ± 10.8	31.7 ± 9.7	27/34	24/27		The judgment method 5
Jianrong Zhao	China	2015	RCT	BA	Tiopronin	31	30	NA	NA	23/8	21/9		The judgment method 3
Xiaopeng Hu	China	2012	RCT	BA	Tiopronin	40	40	39.6		58/22			Undescribed
Shanshan Wei	China	2019	RCT	BA	DG	37	35	51.25 ± 8.07	52.32 ± 7.95	25/12	22/13		The judgment method 2
Yejing Chen	China	2022	RCT	MI	CGAM	51	51	47.3 ± 1.9		59/43			The judgment method 1
Shengting Ruan	China	2017	RCT	MI	RG	37	37	48.2 ± 1.4		14/60			The judgment method 6
Zhibin Yang	China	2021	RCT	MI	PPPC	25	25	40.4 ± 18.58	43.44 ± 17.94	16/9	15/10		The judgment method 1
Lina Tang	China	2012	RCT	MI	Tiopronin	84	28	32.99 ± 12.89	34.71 ± 13.35	51/23	16/12		The judgment method 7
Xuemiao Zhang	China	2011	RCT	MI	RG	47	47	51.4		46/48			The judgment method 1
Jianguo Jiang	China	2010	RCT	MI	RG	32	30	43.5 ± 7.8	44.1 ± 8.3	21/11	18/12		ALT, TBIL, AST, AR, ER 4
Xinzhi Guo	China	2013	RCT	MI	Tiopronin	84	28	32.99 ± 12.89	34.71 ± 13.35	51/23	16/12		The judgment method 7
Guangde Liu	China	2014	RCT	MI	RG	36	36	63.1 ± 1.5		52/20			The judgment method 8
Naqiong Wu	China	2017	RCT	BA	PPPC	79	78	NA	NA	63/16	63/15		The judgment method 7
Majid Marjani	Iran	2018	RCT	Silymarin	Placebo	27	27	52 ± 4	57 ± 4	13/14	12/15		The judgment method 9
Jieting Tang	China	2022	RCT	BA	PPPC	81	78	45.1 ± 15.5	43.5 ± 14.6	43/38	45/33		The judgment method 10
Tehran	Iran	2017	RCT	Silymarin	FA	29	26	53 ± 42	47 ± 41	18/11	16/10		The judgment method 3
Yongfeng Wang	China	2019	RCT	MI	Tiopronin	59	59	39.88 ± 14.7	34.05 ± 11.94	39/20	43/16		The judgment method 3

The judgment method 1:the judgment criteria for 3 types of DILI are: (1) Liver cell injury type: ALT ≥ 3 × ULN, and *R* ≥ 5; (2) Cholestasis type: ALP ≥ 2 × ULN, and *R* ≤ 2; (3) Hybrid type: ALT ≥ 3 × ULN, ALP ≥ 2 × ULN, and 2 < *R* < 5. If ALT and ALP fail to meet the above standards, it is called “abnormal liver biochemistry examination.” R = (ALT measured value/ALT ULN)/(ALP measured value/ALP ULN). The judgment method 2:according to the “Clinical Diagnosis and Treatment Guidelines for Digestive System Diseases” (1), there is a history of using antituberculosis drugs that may cause liver damage, and the occurrence time is consistent with the incidence pattern of DILI. Most liver damage occurs 5 days to 2 months after the use of antituberculosis drugs, and those with specific heterogeneous reactions can occur within 5 days. (2) Clinical process: The rapid recovery of abnormal liver biochemical indicators after discontinuation of medication indicates the occurrence of DILI. Patients with liver cell injury have a high level of ALT peak levels that decrease by > 50% within 8 days, and a significant decrease of ≥ 50% within 30 days; It is important to note that the peak level of serum ALP or total bilirubin in patients with cholestasis decreases by ≥ 50% within 180 days. (3) Liver damage caused by other causes or diseases must be excluded. (4) Positive reaction after re medication: Liver function damage occurs after re medication. If it meets the above diagnostic criteria (1), (2), and (3), or if 2 of the first 3 items meet the criteria, plus (4), it can be diagnosed as ATB-DILI. The judgment method 3: the evaluation criteria for drug-induced liver injury: according to the causal relationship evaluation criteria table for acute drug-induced liver injury (RUCAM simplified scoring system), the score is greater than 6 points. The judgment method 4: the diagnosis of liver injury is based on the WHO classification criteria for adverse reactions to anticancer drugs. The judgment method 5: liver function indicators in line with alanine aminotransferase < 5 × upper limit of normal value (ULN), aspartate transferase <5 ULN, total bilirubin <3 ULN.The judgment method 6: see “Diagnosis and Treatment of Modern Liver Diseases.” The judgment method 7: according to the diagnostic criteria for digestive disease week (DDW) drug-induced liver injury developed by the DDW in Japan in 2004, the score is >6 (scored by a 3-person expert group). The judgment method 8: drug induced liver disease: (1) There is a history of using harmful drugs before the onset of the disease, and the cause and effect of the disease are obvious in a chronological order after the use of drugs. Liver damage occurs within 1–4 weeks. (2) Obvious gastrointestinal symptoms and abnormal liver function. (3) Pathological phenomenon of liver parenchymal cell damage and intrahepatic cholestasis. (4) After discontinuation of medication, symptoms, signs, and liver damage improve or recover. If similar drugs are used again, it can cause liver damage symptoms. (5) Eliminate viral hepatitis and other liver diseases. (6) Macrophage or lymphoblastic transformation test is positive. If the first item above is added to any 2 of items 2–6, the diagnosis will be established. The judgment method 9: see “Internal Medicine 7th Edition.” The judgment method 10: DILI was defined as (1) increasing of AST or ALT to 3 times more than upper limit of normal (40 IU/L), concomitant with symptoms of hepatic toxicity consisting nausea, vomiting, anorexia, weakness and abdominal pain. (2) rise in AST or ALT more than 5 times or total serum bilirubin more than 2 mg/dl.

ALP = alkaline phosphatase; AR = adverse reactions; BA = bicyclic alcohol; CGAM = compound glycyrrhizic acid monoamine; CG = compound glycyrrhizin; DG = diammonium glycyrrhizinate; ER = effective rate; FA = folic acid; RT = routine treatment; HGPF = hepatocyte growth-promoting factor; MI = magnesium isoglycyrrhizinate; PPPC = polyene phosphatidylcholine; RG = reduced glutathione.

### 3.3. Network meta-analysis results

#### 3.3.1. Evidence network.

**Figure 3.**
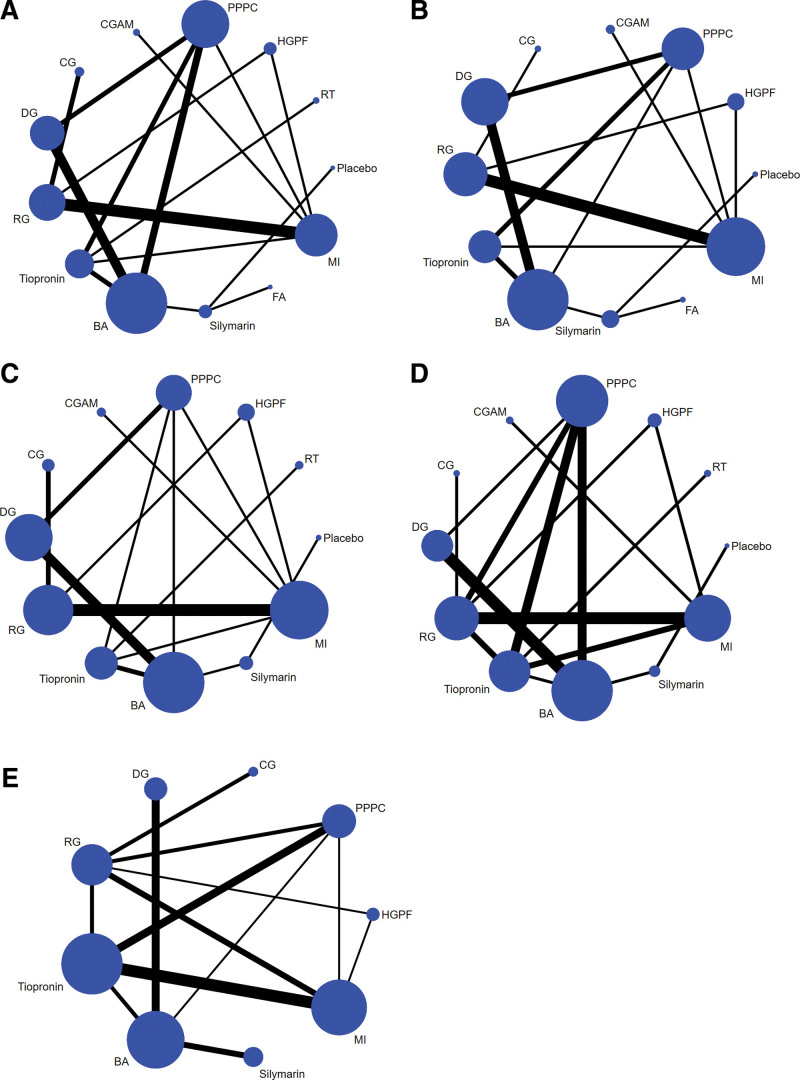
(A) Network evidence map of ALT. (B) Network evidence map of AST. (C) Network evidence map of TBIL. (D) Network evidence map of adverse reactions. (E) Network evidence map of effective rate.

In this network meta-analysis, 13 drugs were involved, and the types of drugs are detailed in Table 2 below. The network evidence graph of ALT, AST, TBIL, adverse reactions, and effective rate shows direct and indirect evidence among diverse intervention measures, which provided the fundamental network meta-analysis conditions (Fig. 3).

**Table 2:** The comparative results of network meta-analysis between ALT under different interventions.

Placebo												
−22.31 (−80.86, 36.25)	RT											
−10.85 (−72.40, 50.71)	11.46 (−42.48, 65.40)	HGPF										
−19.07 (−67.01, 28.87)	3.24 (−33.84, 40.32)	−8.22 (−49.30, 32.86)	PPPC									
−11.46 (−75.20, 52.29)	10.85 (−45.44, 67.15)	−0.61 (−45.00, 43.78)	7.61 (−36.51, 51.73)	CGAM								
7.38 (−53.42, 68.18)	29.69 (−23.44, 82.82)	18.23 (−19.31, 55.77)	26.45 (−13.56, 66.47)	18.84 (−24.58, 62.26)	CG							
−17.78 (−65.72, 30.16)	4.53 (−34.44, 43.49)	−6.93 (−50.90, 37.03)	1.28 (−15.78, 18.35)	−6.33 (−53.16, 40.51)	−25.17 (−68.13, 17.80)	DG						
−12.05 (−68.21, 44.10)	10.25 (−37.43, 57.94)	−1.21 (−30.51, 28.09)	7.01 (−25.42, 39.44)	−0.60 (−37.13, 35.94)	−19.44 (−42.91, 4.03)	5.73 (−30.30, 41.75)	RG					
−22.04 (−70.93, 26.86)	0.27 (−32.37, 32.92)	−11.19 (−54.16, 31.78)	−2.97 (−20.62, 14.68)	−10.58 (−56.47, 35.31)	−29.42 (−71.37, 12.53)	−4.25 (−25.61, 17.10)	−9.98 (−44.78, 24.82)	Tiopronin				
3.74 (−42.21, 49.69)	26.05 (−11.23, 63.32)	14.59 (−28.47, 57.64)	22.80 (7.55, 38.06)	15.19 (−30.78, 61.17)	−3.65 (−45.67, 38.38)	21.52 (7.07, 35.97)	15.79 (−19.11, 50.69)	25.77 (7.67, 43.87)	BA			
0.76 (−34.91, 36.44)	23.07 (−25.69, 71.83)	11.61 (−41.26, 64.49)	19.83 (−15.27, 54.93)	12.22 (−43.12, 67.56)	−6.62 (−58.64, 45.40)	18.55 (−16.37, 53.46)	12.82 (−33.66, 59.30)	22.80 (−13.61, 59.22)	−2.97 (−34.91, 28.96)	Silymarin		
9.96 (−38.68, 58.59)	32.27 (−26.67, 91.20)	20.81 (−41.58, 83.19)	29.02 (−19.22, 77.27)	21.41 (−43.07, 85.90)	2.57 (−59.09, 64.23)	27.74 (−20.37, 75.85)	22.01 (−35.05, 79.07)	31.99 (−17.22, 81.21)	6.22 (−39.78, 52.22)	9.19 (−23.91, 42.30)	FA	
10.34 (−44.01, 64.69)	32.65 (−12.71, 78.01)	21.19 (−8.10, 50.48)	29.41 (0.51, 58.31)	21.80 (−11.55, 55.15)	2.96 (−24.85, 30.76)	28.12 (−4.77, 61.02)	22.40 (7.47, 37.32)	32.38 (0.84, 63.92)	6.61 (−25.06, 38.27)	9.58 (−34.60, 53.76)	0.38 (−54.82, 55.59)	MI

BA = bicyclic alcohol; CGAM = compound glycyrrhizic acid monoamine; CG = compound glycyrrhizin; DG = diammonium glycyrrhizinate; ER = effective rate; FA = folic acid; HGPF = hepatocyte growth-promoting factor; MI = magnesium isoglycyrrhizinate; PPPC = polyene phosphatidylcholine; RG = reduced glutathione; RT = routine treatment.

#### 3.3.2. Inspection of consistency.

The model inconsistency test analysis between ALT, AST, TBIL, adverse reactions, and effectiveness showed *P* > .05, indicating insignificant overall inconsistency. Thereafter, we utilized the node-splitting approach for testing local inconsistency, which revealed the absence of inconsistency in direct versus indirect comparisons on ALT, AST, TBIL, adverse reactions, and effective rate (*P* > .05), accompanied by favorable local consistency. Consequently, we conducted a consistency model analysis.

#### 3.3.3. Network meta-analysis.

*Alanine aminotransferase.* ALT was reported in 27 studies. According to our network meta-analysis, ALT levels in cases receiving polyene phosphatidylcholine (PPPC) and diammonium glycyrrhizinate (DG) treatment increased compared with those in patients receiving bicyclic alcohol (BA) treatment, and those in patients after PPPC and reduced glutathione (RG) treatments increased relative to those receiving magnesium isoglycyrrhizinate (MI) treatment, with significant differences (*P* < .05). Besides, ALT was not significantly different between the other 2 interventions (*P* > .05) (Table 2).

**Figure 4.**
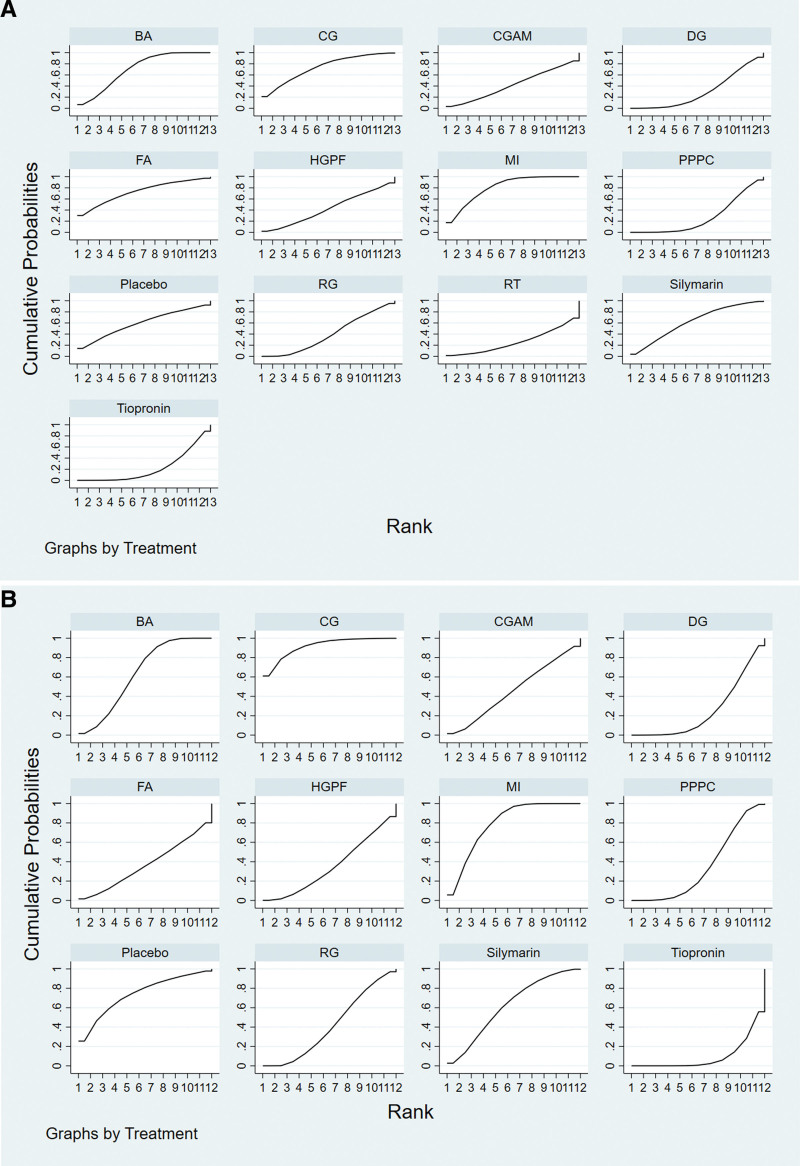
(A) SUCRAT graph of ALT. (B) SUCRAT graph of AST. (C) SUCRAT graph of TBIL. (D) SUCRAT graph of adverse reactions. (E) SUCRAT graph of effective rate.

SUCRA analysis showed that the ALT levels followed the order of MI (81.1%) >  compound glycyrrhizin (CG) (73.4%) > folic acid (72.9%) > BA (71%) > Silymarin (62%) > Placebo (59.5%) >  hepatocyte growth-promoting factor (HGPF) (42.7%) >  compound glycyrrhizic acid monoamine (CGAM) (42.1%) > RG (39.8%) > DG (30.4%) > PPPC (27.1%) > routine treatment (RT) (26%) > Tiopronin (22%), indicating that MI was the possible intervention that had minimum ALT post-treatment (Fig. 4A).

*Aspartate aminotransferase.* AST was reported in 23 studies. Based on our network meta-analysis, AST levels in cases receiving PPPC and HGPF treatments increased compared with those in patients receiving CG treatment, those in patients after tiopronin and DG treatments elevated relative to those in patients receiving BA, and those in patients after PPPC, RG, tiopronin and DG treatments increased compared with those in patients receiving MI treatment, with significant differences (*P* < .05). Besides, AST was compared between the other 2 intervention measures, but no significant difference was detected (*P* > .05) (Table 3).

**Table 3:** The comparative results of network meta-analysis between AST under different interventions.

Placebo											
−24.32 (−79.93, 31.28)	HGPF										
−24.27 (−69.87, 21.33)	0.05 (−33.73, 33.84)	PPPC									
−18.36 (−75.46, 38.74)	5.96 (−31.25, 43.18)	5.91 (−30.22, 42.04)	CGAM								
15.50 (−43.11, 74.11)	39.82 (2.37, 77.27)	39.77 (1.19, 78.35)	33.86 (−7.75, 75.47)	CG							
−28.88 (−73.76, 16.00)	−4.56 (−41.01, 31.89)	−4.61 (−20.08, 10.85)	−10.52 (−49.16, 28.11)	−44.38 (−85.31, −3.45)	DG						
−20.70 (−72.16, 30.76)	3.62 (−21.10, 28.34)	3.57 (−22.85, 29.98)	−2.34 (−33.01, 28.32)	−36.20 (−64.34, −8.07)	8.18 (−21.57, 37.93)	RG					
−37.69 (−83.62, 8.24)	−13.36 (−48.20, 21.48)	−13.42 (−28.64, 1.81)	−19.33 (−56.44, 17.79)	−53.19 (−92.69, 13.68)	−8.80 (−27.04, 9.44)	−16.98 (−44.74, 10.77)	Tiopronin				
−10.76 (−53.97, 32.45)	13.57 (−22.51, 49.65)	13.51 (−1.87, 28.90)	7.60 (−30.68, 45.89)	−26.26 (−66.86, 14.35)	18.13 (5.57, 30.69)	9.95 (−19.35, 39.24)	26.93 (10.73, 43.13)	BA			
−10.15 (−44.44, 24.14)	14.17 (−30.94, 59.29)	14.12 (−17.36, 45.59)	8.21 (−38.71, 55.12)	−25.65 (−74.44, 23.14)	18.73 (−11.55, 49.02)	10.55 (−29.34, 50.44)	27.53 (−4.38, 59.45)	0.60 (−27.03, 28.24)	Silymarin		
−23.44 (−67.83, 20.95)	0.88 (−52.33, 54.09)	0.83 (−41.44, 43.10)	−5.08 (−59.83, 49.67)	−38.94 (−95.30, 17.42)	5.44 (−35.95, 46.84)	−2.74 (−51.60, 46.12)	14.25 (−28.35, 56.84)	−12.68 (−52.18, 26.81)	−13.29 (−41.51, 14.93)	FA	
−0.97 (−50.86, 48.93)	23.36 (−1.35, 48.07)	23.30 (0.24, 46.36)	17.39 (−10.43, 45.22)	−16.47 (−47.41, 14.48)	27.92 (1.09, 54.74)	19.73 (6.85, 32.62)	36.72 (12.15, 61.29)	9.79 (−16.53, 36.11)	9.18 (−28.61, 46.98)	22.47 (−24.70, 69.64)	MI

BA = bicyclic alcohol; CGAM = compound glycyrrhizic acid monoamine; CG = compound glycyrrhizin; DG = diammonium glycyrrhizinate; FA = folic acid; HGPF = hepatocyte growth-promoting factor; MI = magnesium isoglycyrrhizinate; PPPC = polyene phosphatidylcholine; RG = reduced glutathione.

Based on SUCRA results, the AST levels satisfied the order of CG (91.6%) > MI (79.1%) > placebo (74.1%) > BA (63.7%) > silymarin (61.9%) > CGAM (45.9%) > RG (41.4%) > HGPF (35.3%) > PPPC (35.1%) >  folic acid (26.9%) > DG (25.2%) > tiopronin (9.8%), demonstrating that CG was the potential intervention that had minimum AST levels post-treatment (Fig. 4B).

*Total bilirubin.* TBIL was reported in 23 studies. Based on our network meta-analysis, TBIL levels in cases receiving RT treatment were elevated relative to those in cases receiving Placebo, HGPF, PPPC, CGAM, CG, DG, RG, tiopronin, BA, silymarin, and MI treatments; those in patients after PPPC and RG treatments increased compared with those in cases receiving MI treatment, with significant difference (*P* < .05). Moreover, TBIL levels were compared between the other 2 intervention measures, which revealed no significant difference (*P* > .05) (Table 4).

**Table 4:** The comparative results of network meta-analysis between TBIL under different interventions.

Placebo											
−81.49 (−103.61, −59.38)	RT										
−0.92 (−19.36, 17.51)	80.57 (60.15, 100.99)	HGPF									
4.69 (−10.11, 19.48)	86.18 (68.57, 103.79)	5.61 (−6.57, 17.79)	PPPC								
−0.26 (−19.34, 18.83)	81.24 (60.25, 102.22)	0.67 (−13.23, 14.57)	−4.94 (−18.04, 8.16)	CGAM							
1.04 (−17.43, 19.50)	82.53 (62.08, 102.98)	1.96 (−10.17, 14.10)	−3.65 (−15.87, 8.58)	1.29 (−12.66, 15.25)	CG						
1.70 (−12.70, 16.10)	83.20 (65.40, 101.00)	2.63 (−10.35, 15.60)	−2.98 (−8.81, 2.84)	1.96 (−11.89, 15.81)	0.66 (−12.36, 13.69)	DG					
−0.78 (−17.49, 15.92)	80.71 (61.84, 99.58)	0.14 (−9.09, 9.37)	−5.47 (−14.82, 3.88)	−0.53 (−12.04, 10.98)	−1.82 (−9.71, 6.06)	−2.49 (−12.86, 7.88)	RG				
−1.49 (−16.38, 13.39)	80.00 (63.64, 96.36)	−0.57 (−12.79, 11.65)	−6.18 (−12.71, 0.35)	−1.24 (−14.39, 11.91)	−2.53 (−14.81, 9.74)	−3.20 (−10.22, 3.82)	−0.71 (−10.13, 8.70)	Tiopronin			
0.42 (−13.29, 14.14)	81.92 (64.45, 99.38)	1.35 (−11.42, 14.12)	−4.26 (−10.21, 1.69)	0.68 (−12.98, 14.34)	−0.61 (−13.43, 12.21)	−1.28 (−5.94, 3.39)	1.21 (−8.90, 11.32)	1.92 (−4.20, 8.04)	BA		
0.51 (−9.41, 10.44)	82.01 (61.88, 102.14)	1.44 (−14.68, 17.55)	−4.17 (−15.80, 7.46)	0.77 (−16.07, 17.61)	−0.52 (−16.67, 15.62)	−1.19 (−12.27, 9.89)	1.30 (−12.80, 15.39)	2.01 (−9.73, 13.74)	0.09 (−10.02, 10.20)	Silymarin	
2.04 (−13.96, 18.05)	83.54 (65.31, 101.76)	2.97 (−6.25, 12.19)	−2.64 (−10.61, 5.33)	2.30 (−8.10, 12.70)	1.01 (−8.29, 10.30)	0.34 (−8.80, 9.49)	2.83 (−2.10, 7.76)	3.54 (−4.50, 11.58)	1.62 (−7.23, 10.47)	1.53 (−11.71, 14.78)	MI

BA = bicyclic alcohol; CGAM = compound glycyrrhizic acid monoamine; CG = compound glycyrrhizin; DG = diammonium glycyrrhizinate; FA = folic acid; HGPF = hepatocyte growth-promoting factor; MI = magnesium isoglycyrrhizinate; PPPC = polyene phosphatidylcholine; RG = reduced glutathione.

SUCRA analysis showed that the TBIL levels satisfied the order of PPPC (83%) > MI (66.2%) > DG (61.9%) > CG (56.9%) > silymarin (54%) > placebo (51.4%) > BA (51.3%) > CGAM (49.4%) > HGPF (45.5%) >RG (43.1%) > tiopronin (37.3%) > RT (0%), indicating that PPPC was the possible intervention that had minimum TBIL level post-treatment (Fig. 4C).

*Adverse reactions*. The adverse events were reported in 23 studies. As revealed by our network meta-analysis, the difference was not significant between the 2 intervention measures (*P* > .05) (Table 5).

**Table 5:** The comparative results of network meta-analysis between adverse reactions under different interventions.

Placebo											
1.34 (0.00, 420.93)	RT										
1.88 (0.02, 165.24)	1.40 (0.02, 92.25)	HGPF									
1.74 (0.03, 112.54)	1.30 (0.02, 68.65)	0.93 (0.18, 4.79)	PPPC								
1.27 (0.02, 99.61)	0.95 (0.02, 54.63)	0.68 (0.12, 3.84)	0.73 (0.20, 2.66)	CGAM							
3.01 (0.04, 229.67)	2.25 (0.04, 127.70)	1.60 (0.33, 7.83)	1.73 (0.52, 5.74)	2.37 (0.60, 9.36)	CG						
1.53 (0.02, 100.47)	1.14 (0.02, 65.78)	0.81 (0.13, 5.14)	0.88 (0.38, 2.05)	1.20 (0.26, 5.59)	0.51 (0.12, 2.19)	DG					
2.26 (0.03, 162.85)	1.69 (0.03, 90.16)	1.20 (0.29, 4.99)	1.30 (0.49, 3.43)	1.78 (0.54, 5.79)	0.75 (0.37, 1.52)	1.48 (0.41, 5.32)	RG				
1.34 (0.02, 91.76)	1.00 (0.02, 49.35)	0.71 (0.16, 3.27)	0.77 (0.37, 1.59)	1.05 (0.35, 3.15)	0.44 (0.15, 1.28)	0.87 (0.29, 2.64)	0.59 (0.27, 1.30)	Tiopronin			
1.58 (0.03, 97.76)	1.18 (0.02, 65.04)	0.84 (0.15, 4.82)	0.91 (0.49, 1.67)	1.24 (0.30, 5.12)	0.52 (0.14, 2.00)	1.03 (0.51, 2.09)	0.70 (0.22, 2.18)	1.18 (0.46, 3.00)	BA		
1.33 (0.33, 5.39)	1.00 (0.00, 264.47)	0.71 (0.01, 50.14)	0.77 (0.02, 39.05)	1.05 (0.02, 65.39)	0.44 (0.01, 26.92)	0.87 (0.02, 45.19)	0.59 (0.01, 33.74)	1.00 (0.02, 54.07)	0.84 (0.02, 41.07)	Silymarin	
2.12 (0.03, 150.03)	1.59 (0.03, 81.62)	1.13 (0.26, 4.86)	1.22 (0.50, 2.96)	1.67 (0.65, 4.24)	0.70 (0.26, 1.93)	1.39 (0.41, 4.69)	0.94 (0.45, 1.94)	1.59 (0.89, 2.82)	1.34 (0.46, 3.90)	1.59 (0.03, 89.33)	MI

BA = bicyclic alcohol; CGAM = compound glycyrrhizic acid monoamine; CG = compound glycyrrhizin; DG = diammonium glycyrrhizinate; ER = effective rate; FA = folic acid; HGPF = hepatocyte growth-promoting factor; MI = magnesium isoglycyrrhizinate; PPPC = polyene phosphatidylcholine; RG = reduced glutathione; RT = routine treatment.

The results of the SUCRA showed that the adverse reaction rate followed the order of placebo (83%) > RT (66.2%) > HGPF (61.9%) > PPPC (56.9%) > CGAM (54%) > CG (51.4%) > DG (51.3%) > RG (49.4%) > tiopronin (45.5%) > BA (43.1%) > Ssilymarin (37.3%) > MI (0%), indicating that placebo was the potential intervention that had minimum adverse reactions post-treatment (Fig. 4D).

*Effective rate.* The incidence of effective rate was reported in 26 studies. According to our network meta-analysis, the effective rate in cases receiving CG treatment increased compared with that in those receiving PPPC treatment, that in patients after HGPF, CG, and RG treatments was higher than that after tiopronin treatment, that in cases receiving BA treatment increased relative to that after PPPC, DG, and Tiopronin treatments, that in cases receiving silymarin treatment elevated compared with that in cases receiving tiopronin treatment, and that in cases receiving MI treatment increased compared with that in those receiving tiopronin and PPPC treatments, with significant differences (*P* < .05). Besides, the effective rate was compared between the other 2 intervention measures, which revealed no significant difference (*P* > .05) (Table 6).

**Table 6:** The comparative results of network meta-analysis between effective rate under different interventions.

HGPF								
1.17 (0.95, 1.43)	PPPC							
0.94 (0.75, 1.18)	0.81 (0.66, 0.99)	CG						
1.06 (0.80, 1.41)	0.91 (0.73, 1.14)	1.13 (0.84, 1.51)	DG					
1.01 (0.85, 1.19)	0.86 (0.75, 1.00)	1.07 (0.92, 1.24)	0.95 (0.74, 1.22)	RG				
1.28 (1.06, 1.55)	1.10 (0.97, 1.24)	1.36 (1.12, 1.66)	1.21 (0.97, 1.51)	1.27 (1.12, 1.45)	Tiopronin			
0.85 (0.66, 1.11)	0.73 (0.60, 0.89)	0.91 (0.70, 1.18)	0.81 (0.72, 0.91)	0.85 (0.68, 1.06)	0.67 (0.55, 0.81)	BA		
0.95 (0.71, 1.26)	0.81 (0.64, 1.02)	1.00 (0.75, 1.35)	0.89 (0.75, 1.06)	0.94 (0.73, 1.21)	0.74 (0.59, 0.93)	1.11 (0.98, 1.25)	Silymarin	
0.90 (0.77, 1.06)	0.77 (0.67, 0.89)	0.96 (0.80, 1.15)	0.85 (0.67, 1.09)	0.90 (0.80, 1.00)	0.70 (0.63, 0.79)	1.06 (0.85, 1.31)	0.96 (0.74, 1.23)	MI

BA = bicyclic alcohol; CGAM = compound glycyrrhizic acid monoamine; CG = compound glycyrrhizin; DG = diammonium glycyrrhizinate; ER = effective rate; FA = folic acid; HGPF = hepatocyte growth-promoting factor; MI = magnesium isoglycyrrhizinate; PPPC = polyene phosphatidylcholine; RG = reduced glutathione; RT = routine treatment.

SUCRA analysis showed that the effective rate followed the order of HGPF (83%) > PPPC (66.2%) > CG (61.9%) > DG (56.9%) > RG (54%) > tiopronin (51.4%) > BA (51.3%) > silymarin (49.4%) >MI (45.5%), indicating that HGPF was the potential intervention measure that had highest effective rate post-treatment (Fig. 4E).

### 3.4. Publication bias

**Figure 5.**
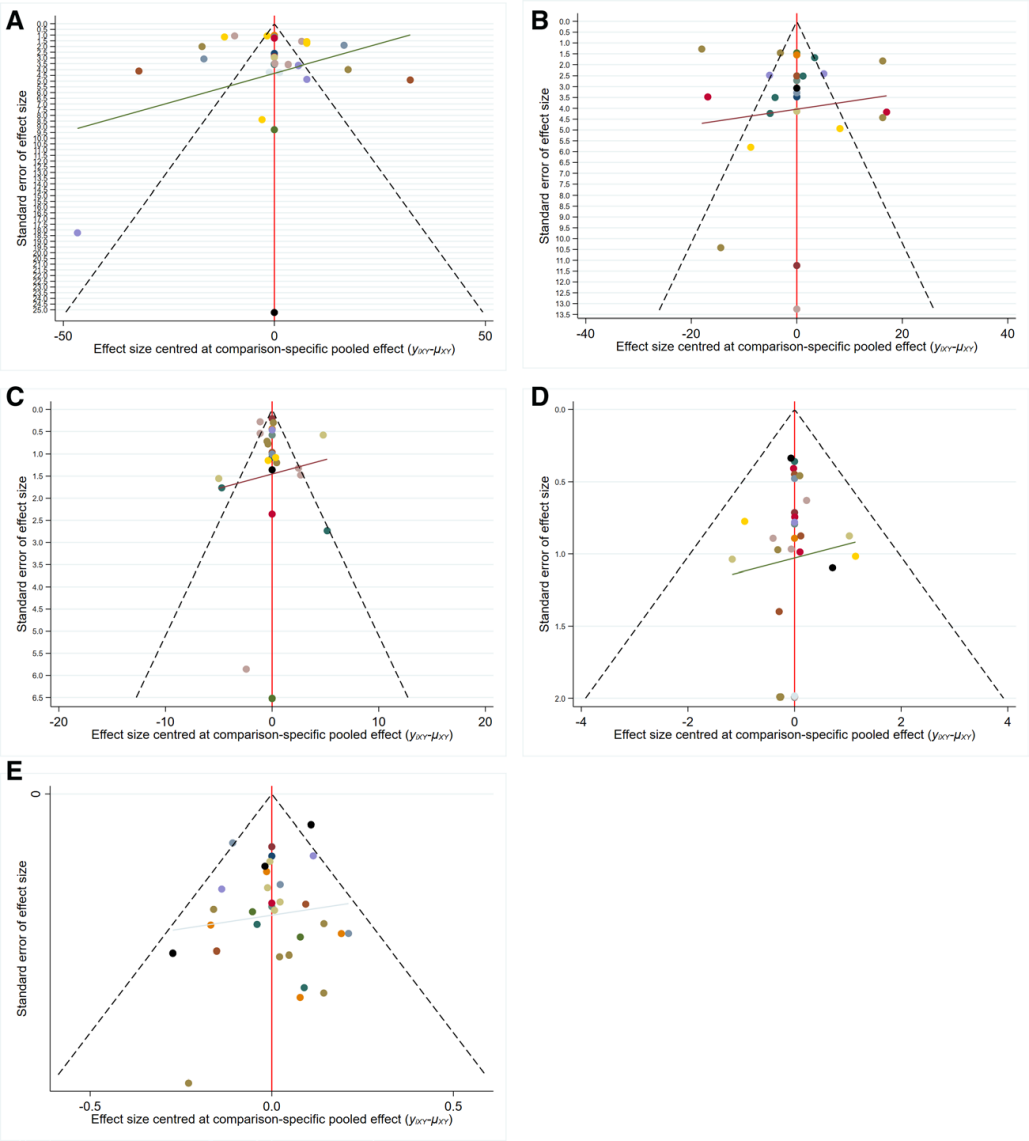
(A) The funnel plot of ALT. (B) The funnel plot of AST. (C) The funnel plot of TBIL. (D) The funnel plot of adverse reactions. (E) The funnel plot of effective rate.

Based on the funnel chart for ALT, AST, TBIL, adverse reactions, and effective rate, all points exhibited scattered distribution and incomplete symmetry, indicating the presence of a certain publication bias. Besides, some points showed scattered distribution at the funnel chart bottom in every research index, demonstrating the presence of a small sample effect (Fig. 5).

## 4. Discussion

DILI is the most common and serious adverse drug reaction that can cause liver failure and even lead to death.^[41]^ Currently, DILI is still a major unresolved global problem, and there are many types of drugs available for the treatment of DILI. As shown in previous studies, diammonium glycyrrhizin can improve DILI and rapidly reduce the level of alanine transaminase.^[42]^ Besides, compound glycyrrhizin exhibits the activities of anti-inflammation and hormone-like activities, shows protective effects on the liver cell membrane, decreases phospholipase A2 activity, and alleviates liver tissue damage.^[43]^ However, due to the complex condition of DILI patients, each drug may have its own limitations. Consequently, the present work applied network meta-analysis in evaluating the safety and therapeutic effects of 13 diverse liver protective drugs for DILI cases. Some studies show that liver biopsy analysis in suspected DILI cases helps doctors better determine whether the liver injury is caused by drugs or other reasons, which facilitates the next step of clinical treatment.^[1]^ However, at present, traditional liver biomarkers such as ALT, AST, TBIL, and alkaline phosphatase are still used as the clinical indicators of liver injury. As revealed by the analysis results of 32 clinical studies, MI was the potential intervention that showed minimum ALT level post-treatment. CG was the potential intervention that exhibited minimum ALT level post-treatment. PPPC was the potential intervention that displayed minimum TBIL post-treatment. Placebo was the potential intervention that displayed minimum adverse reactions post-treatment, followed by RT. HGPF might be the intervention with the highest effective rate after treatment.

The limitations of this study are as follows: some studies have a moderate to high risk of bias, and most studies have not reported random allocation and hidden allocation methods, which may have an impact on the conclusions. Most of the studies were conducted in China. This could be a geographical bias.

To sum up, the present work compared the clinical effects of 13 liver protective drugs through meta-analysis and provided a systematic understanding of commonly used drugs for the treatment of DILI in clinical practice. Plenty of direct and circumstantial evidence was evaluated at the same time, and the research results were relatively objective and accurate, which enabled the healthcare system decision-makers, healthcare workers, and nursing specialists to choose among the multiple alternative options rather than based on single cognitive intervention research or traditional meta-analysis.

## Author contributions

**Funding acquisition and visualization:** Anhao Wu.

**Writing—review & editing:** Anhao Wu, Yifan Wang.

**Data curation:** Chengcheng Li, Xin Yang.

**Formal analysis and Writing—original draft:** Chengcheng Li.

**Investigation and software:** Yuhang Quan.

**Methodology:** Xin Yang.

**Conceptualization and supervision:** Yifan Wang.

## References

[1] Ahmad J Barnhart HX Bonacini M.; Drug-Induced Liver Injury Network. Value of liver biopsy in the diagnosis of drug-induced liver injury. J Hepatol. 2022;76:1070–8. 35074471 10.1016/j.jhep.2021.12.043 PMC9018618

[2] Worland T Chin KL Rodrigues B. A retrospective case-controlled cohort study of inpatient drug induced liver injury: the RIDDLE study. Transl Gastroenterol Hepatol. 2020;5:33. 32632384 10.21037/tgh.2019.10.15 PMC7063506

[3] Shen T Liu Y Shang J. Incidence and etiology of drug-induced liver injury in Mainland China. Gastroenterology. 2019;156:2230–41.e11. 30742832 10.1053/j.gastro.2019.02.002

[4] Wang J Song H Ge F. Landscape of DILI-related adverse drug reaction in China Mainland. Acta pharmaceutica Sinica B. 2022;12:4424–31. 36561993 10.1016/j.apsb.2022.04.019 PMC9764066

[5] Fontana RJ Liou I Reuben A. AASLD practice guidance on drug, herbal, and dietary supplement-induced liver injury. Hepatology. 2023;77:1036–65. 35899384 10.1002/hep.32689 PMC9936988

[6] Björnsson ES. Epidemiology, predisposing factors, and outcomes of drug-induced liver injury. Clin Liver Dis. 2020;24:1–10. 31753242 10.1016/j.cld.2019.08.002

[7] McAtee C. DNP, CCRN, ACNP-BC, CNE. Drug-induced liver injury. Crit Care Nurs Clin North Am. 2022;34:267–75. 36049846 10.1016/j.cnc.2022.04.007

[8] Moher D Shamseer L Clarke M.; PRISMA-P Group. Preferred reporting items for systematic review and meta-analysis protocols (PRISMA-P) 2015 statement. Syst Rev. 2015;4:1. 25554246 10.1186/2046-4053-4-1 PMC4320440

[9] Hujun L Liping G. Comparison of effects on drug-induced liver injury treated with three common liver drugs. J Kunming Med Univ. 2017;38:94–7.

[10] Zhun W Tianfu G. Efficacy of three liver-protecting drugs commonly used in treatment of drug-induced liver injury. J CIin Hepaltol. 2016;32:761–3.

[11] Hong D Zhuo T Lianning W. Clinical analysis of 3 in 126 HIV/TB patients. J Clin Pulm Med. 2012;32:1256–8.

[12] Rongxia L Jie L Linglin Z. Effect and economic comparison among different liver protective drugs in treatment of anti-rheumatoid drug-induced liver injury. China Med. 2019;14:369–99.

[13] Jiao Y Wei W. Effects of polyene phosphatidylcholine on serum NO and NOS levels in patients with acute liver injury. Chin Hepatol. 2018;23:269–70.

[14] Yunhua L Rongfeng D Hubo X. Therapeutic efficacy of polyene phosphatidylcholine in patients with anti-tuberculosis agents-induced liver injury. J Prac Hepatol. 2021;24:228–31.

[15] Fuping Y Ming L. Observation of therapeutic effect of polyene phosphatidylcholine on drug-induced liver injury. Int J Lab Med. 2014;35:1952–53.

[16] Huajun W. Clinical analysis of effects of compound glycyrrhizin in the treatment of patients with antituberculosis drug-induced hepatitis. J Clin Pulm Med. 2010;15:496–7.

[17] Xianfeng G. Effect of diammonium glycyrrhizin and polyene phosphatidylcholine on drug induced liver injury induced by anti tuberculosis of pulmonary tuberculosis. Shanxi Med J. 2021;50:3275–8.

[18] Yongjun Y Hui Z Jinshan Z. Clinical observation on the treatment of 46 cases of anti tuberculosis drug induced liver injury with tiopronin. J CIinHepaltol. 2010;11:298–9.

[19] Jianguo L Hui G Junlin Y. Effects of bicyclol on serum MDA and SOD activities in patients with liver injury induced by antipsychotic drugs. Chin J Lab Diagn. 2014;18:1473–5.

[20] Fei L Yingge Z Desheng L. Inhibition effect of bicyclol on lipid peroxidation in liver injury induced by neuropathic drugs. Med J West China. 2018;30:446–8.

[21] Houxiong L Xiuniu H Zhide W. Comparison of the therapeutic effects of bicyclol and polyene Phosphatidylcholine on antituberculosis drug-induced liver injury. Shandong Med J. 2019;59:69–71.

[22] Xiaoyong Z Xueyuan Y Yunyun S. Bicyclol versus diammonium glycyrrhizinate for the treatment of drug-induced hepatic injury in patients with severe psoriasis: a randomized controlled trial. Chin J Dermatol. 2015;48:245–7.

[23] Yun T Yiye W. Therapeutic and prophyiatic effect of bicyciol on drug–induced liver injury for lung cancer patients. J Med Res. 2011;41:156–8.

[24] Lin Z Yongsu W. Efficacy of bicyclol tablets vs Silymarin capsules in the treatment of anti-tuberculosis drug-induced liver injury. Eval Anal Drug Use Chin Hosp. 2015;15:519–21.

[25] Jianrong Z Gancheng P Ronghui X. Clinical observation on the therapeutic effect of bicyclic alcohol tablets on antiviral therapy related drug-induced liver injury in chronic HIV infected individuals. Chin Hepatol. 2015;20:798–802.

[26] Xiaopeng H Yin H Wei W. Therapeutic efficacy of bicyclol for the drug induced hepatic injury after renaI transpIantation. Chin J Gastroenterol Hepatal. 2012;21:342–4.

[27] Shanshan Z Yongmei L Fang L. Clinical effect of bicyclol in treatment of patients with liver injury induced by anti-tuberculosis drugs. Med Pharm J Chin PLA. 2019;31:47–50.

[28] Yejing C Qinping L Shaoying G. Comparison of therapeutic efficacy of magnesium isoglycyrrhizinate and compound glycyrrhizin monoamine in treatment of patients with drug-induced liver injury. J Prac Hepatol. 2022;25:371–4.

[29] Shengting R Guangyun X. Appfication of glycyrrhizic acid magnesium and reduced glutathinone in treatment of patients with anti-rheumatoid arthritis-induced fiver injuries Ruan. J Prac Hepatol. 2017;20:608–9.

[30] Zhibin Y Yanfang S Li pan. Efficacy of magnesium isoglycyrrhizinate in anti-tuberculosis drug-induced liver injury. Clin Focus. 2021;36:972–5.

[31] Lina T Feng L Zang S. Magnesium isoglycyrrhizinate used in the treatment of chemotherapeutic drugs-induced acute liver dysfunction: a phaseIII clinical trial. Tumor. 2012;32:738–43.

[32] Xuemiao Z Xin Z Xiushan R. Clinical observation of magnesium isoglycyrrhizinate injection preventing acute liver injury induced by chemotherapy drugs. China Pharm. 2011;22:3218–20.

[33] Jianguo J. Clinical observation of magnesium isoglycyrrhizinate injection in the treatment of drug-induced liver injury. Chin J Postgard Med. 2010;33:72–3.

[34] Xinzhi G Yu C Junwei C. Randomized double-blinded and active drug-controlled clinical study of Magnesium Isoglycyrrhizinate Injection in the treatment of antituberculosis drug-induced acute hepatic dysfunction. J Microbes Infect. 2013;8:157–62.

[35] Guangde L Yao H Fangyan Z. Clinical analysis of magnesium iso glycyrrhizin injection in the treatment of drug-induced liver injury. Mod J Integr Tradit Chin West Med. 2014;23:2680–1.

[36] Wu N Wang L Han Z. A multicenter and randomized controlled trial of bicyclol in the treatment of statin-induced liver injury. Med Sci Monit. 2017;23:5760–6. 29200411 10.12659/MSM.904090 PMC5728082

[37] Majid M Fanak F Makan S. Evaluation of silymarin for management of anti-tuberculosis drug induced liver injury: a randomized clinical trial. Gastroenterol Hepatol Bed Bench. 2019;12:138–42. 31191838 PMC6536020

[38] Jieting T Jin G Naihui C. Efficacy and safety of bicyclol for treating patients with idiosyncratic acute drug-induced liver injury: a multicenter, randomized, phase II trial. Liver Int. 2022;42:1803–13. 35567757 10.1111/liv.15290

[39] Masoumeh A Mamak S Mahdi S. Comparison of efficacy of folic acid and silymarin in the management of antiepileptic drug induced liver injury: a randomized clinical trial. Hepatobiliary Pancreat Dis Int. 2017;16:296–302. 28603098 10.1016/s1499-3872(16)60142-x

[40] Yongfeng W Zhenghua W Mengqiu G. Efficacy and safety of magnesium isoglycyrrhizinate injection in patients with acute drug-induced liver injury: a phase II trial. Liver Int. 2019;39:2102–11. 31379118 10.1111/liv.14204

[41] Li X Tang J Mao Y. Incidence and risk factors of drug-induced liver injury. Liver Int. 2022;42:1999–2014. 35353431 10.1111/liv.15262

[42] Tao B Wang G Yin Z. Determination of the contents of antioxidants and their degradation products in sodium chloride injection for blood transfusion. J Anal Methods Chem. 2020;2020:576. 10.1155/2020/8869576 PMC7327558 32655966

[43] Wang L Wen X Hao D. Combination therapy with salicylic acid chemical peels, glycyrrhizin compound, and vitamin C for Riehl’s melanosis. J Cosmet Dermatol. 2020;19:1377–80. 31524950 10.1111/jocd.13153

